# Editorial: Targeting the Wnt/β-catenin signaling pathway in cancer

**DOI:** 10.3389/fonc.2022.1022174

**Published:** 2022-09-13

**Authors:** Simone Patergnani, Donald J. Buchsbaum, Gary A. Piazza

**Affiliations:** ^1^ Department of Medical Sciences, Laboratory for Technologies of Advanced Therapies, University of Ferrara, Ferrara, Italy; ^2^ Department of Radiation Oncology, The University of Alabama at Birmingham, Birmingham, AL, United States; ^3^ Department of Drug Discovery and Development, Harrison College of Pharmacy, Auburn University, Auburn, AL, United States

**Keywords:** Wnt/β-catenin, signaling, targeted therapy, molecular targets, cancer

Wnt/β-catenin signaling, also referred as canonical Wnt signaling, is a highly conserved cellular pathway that regulates multiple biological processes involved in normal physiological and disease processes, including cell proliferation and differentiation, tissue regeneration, migration, autophagy, and apoptosis. The main human disorder associated with dysfunctional Wnt/β-catenin signaling is cancer (1). Indeed, aberrant activation of Wnt/β-catenin signaling resulting from either Wnt overexpression or mutations in pathway components (e.g., APC) that stabilize β-catenin have been extensively reported in gastrointestinal, lung, liver, breast, and prostate cancers, as well as in leukemia and melanoma. Wnt/β-catenin signaling is known to drive Tcf/Lef transcription of proteins essential for cancer cell proliferation and survival, such as cyclin D, Myc, and survivin (2, 3). Wnt/β-catenin signaling also appears to be essential for cancer cell migration and invasion in the case of breast cancer and melanoma (4, 5) and the control of tumor dormancy in colorectal cancers (6). Another important aspect relating to cancer in which Wnt/β-catenin is involved is tumor immunology. Here, Wnt/β-catenin signaling appears to act as a major regulatory mechanism that causes primary resistance to T cell-based immune-oncology therapies (7). Finally, Wnt/β-catenin is involved in the maintenance and propagation of stem cells essential for normal cell turnover as well as tissue regeneration (8, 9). Consequently, Wnt/β-catenin signaling can cause resistance to certain cancer treatments (10), while inhibition can result in toxicity to normal tissues. Considering these aspects, it is not surprising that researchers have proposed multiple molecular strategies to target various nodes within this signaling pathway for cancer treatment. Indeed, the Wnt/β-catenin pathway is complex and embodies a wide number of components, which represent potential targets for cancer treatment or prevention. Furthermore, Wnt/β-catenin may have different roles among cancers of different histologic origins. Therefore, it is necessary to consider all molecular relationships between Wnt/β-catenin signaling and other regulatory pathways in normal tissues and cancer in the development of novel anticancer drugs that target Wnt/β-catenin signaling.

This Research Topic entitled “Targeting the Wnt/β-catenin Signaling Pathway in Cancer” represents a review of the current knowledge of the intrinsic link between Wnt/β-catenin signaling and cancer. The published original research and review articles are briefly described below:

Zhou et al. investigated the molecular mechanisms by which uterine leiomyoma (UL) cells proliferate and unveiled that the specific long noncoding RNA Alu-mediated p21 transcriptional regulator (APTR) was overexpressed in UL human tissues and promoted cell proliferation by increasing the expression of the proteins of the Wnt/β-catenin signaling pathway and by directly interacting with the estrogen receptor alpha (ERα).

Raoul et al. reported the clinical outcome and molecular profile of two cases of metastatic pancreatoblastomas, a rare pancreatic tumor typically found in young children. In this report, they demonstrated that both patients benefited from systemic chemotherapy (FOLFIRINOX regimen). Furthermore, by performing high-throughput sequencing of the tumors analyzed, they found specific mutations of members of the Wnt/β-catenin pathway.

Behrooz and Syahir provides an overview of the state-of-the-art of the interplay between CD133, Wnt/β-catenin and TERT signaling pathways in glioblastoma multiforme (GBM) to demonstrate that this axis may represent a possible therapeutic target against this aggressive cancer type.

She et al. demonstrated that the Fas apoptotic inhibitory molecule 2 (FAIM2) is highly expressed in non-small cell lung cancer (NSCLC) tissues and NSCLC tissues with bone metastasis, and promotes cell proliferation, migration, and invasion, and inhibits cell apoptosis. Interestingly, the authors unveiled that FAIM2 exerts these effects by regulating the Wnt/β-catenin signaling pathway.

Ding et al. identified high expression levels of tumor endothelial marker 8 (TEM8) in samples and cells of lung adenocarcinoma. Such increased levels were associated with tumor size of primary tumor. Mechanistic investigations performed both *in vitro* and *in vivo* showed that TEM8 promoted lung cancer cell proliferation and invasion by activating Wnt/β-catenin signaling.

Ke et al. found that exposure to polycyclic aromatic hydrocarbons (which are widespread environmental pollutants associated with carcinogenicity) suppressed the microRNA (miR), 377-3p, and downregulated the expression of early growth response 1 (EGR1) to facilitate malignant transformation and tumor growth of lung cells by regulating the Wnt/β-catenin pathway.

Zheng et al. demonstrated that the protein A-kinase interacting protein 1 (AKIP1) was elevated in diverse thyroid carcinoma cells and regulated different aspects of tumorigenesis (including apoptosis, cell viability and invasion, and response to chemotherapy) by controlling the activity of PI3K/AKT and β-catenin pathways.

Shah et al. explored the current knowledge of the phosphorylation-dependent regulations of β-catenin and Wnt signaling in cancer providing a detailed overview of the main kinases which regulate the Wnt/β-catenin pathway.

Wang et al. performed a study aimed to investigate the significance of Wnt in the prognosis and tumor immunity in NSCLC and found that WNT2B and WNT7A might have prognostic value in lung adenocarcinoma and in squamous cell lung cancer. Authors performed this investigation by using a publicly available tumor database and validated their findings in NSCLC pathological tissues and cell lines.

## Perspectives

The Wnt/β-catenin signaling pathway was discovered over 30 years ago and has been extensively studied as a key cellular pathway involved in both physiological and pathological processes. While it has been widely reported that Wnt/β-catenin signaling plays a critical role in multiple aspects of tumorigenesis from early stages of malignant transformation to invasion and metastasis, there are no approved anticancer drugs that target this pathway for either the treatment or prevention of cancer. Nonetheless, multiple lines of evidence suggests that targeting the Wnt/β-catenin pathway with small molecule inhibitors can selectively inhibit proliferation and induce apoptosis of cancer cells to inhibit tumor growth. Despite all these aspects, the exact function of Wnt/β-catenin signaling, all of its molecular components, and other intracellular signaling pathways (e.g., RAS/MAPK) that may intersect with Wnt/β-catenin signaling, within the context of normal physiology and cancer biology remain only partially understood. A more complete understanding of these questions holds promise for providing insight to novel molecular targets and the development of efficacious anticancer drugs for a wide range of malignancies that are driven by Wnt/β-catenin signaling.

## Author contributions

All authors listed have made a substantial, direct, and intellectual contribution to the work and approved it for publication.

## Funding

SP was supported by Fondazione Umberto Veronesi and by A-ROSE. DB was supported by the American Board of Obstetsrics & Gynecology, the Norma Livingston Foundation, and the Foundation for Womens Cancer. GP was supported, in part, by the National Cancer Institute of the National Institutes of Health under awards R01CA197147, R01CA254197, and R01CA238514.

## Acknowledgments

We thank all the authors who contributed to this Research Topic as well as various reviewers/editors of the respective manuscripts, for their efforts, timely responses and enthusiasm. We also thank the Frontiers Editorial Office for their assistance and support.

## Conflict of interest

The authors declare that the research was conducted in the absence of any commercial or financial relationships that could be construed as a potential conflict of interest.

## Publisher’s note

All claims expressed in this article are solely those of the authors and do not necessarily represent those of their affiliated organizations, or those of the publisher, the editors and the reviewers. Any product that may be evaluated in this article, or claim that may be made by its manufacturer, is not guaranteed or endorsed by the publisher.
